# An Investigation of the Mechanical Properties and Thermal Insulation of Foamed Rubberised Polypropylene Fibre Concrete That Incorporates a High Quantity of Crumb Rubber Granules

**DOI:** 10.3390/polym17070967

**Published:** 2025-04-02

**Authors:** Zhi Heng Lim, Foo Wei Lee, Kim Hung Mo, Ren Jie Chin, Kim Ho Yeap, Ming Kun Yew

**Affiliations:** 1 Lee Kong Chian Faculty of Engineering and Science, Universiti Tunku Abdul Rahman, Jalan Sungai Long, Bandar Sungai Long, Cheras, Kajang 43000, Selangor, Malaysia; leon2810@1utar.my (Z.H.L.); chinrj@utar.edu.my (R.J.C.); yewmk@utar.edu.my (M.K.Y.); 2 Department of Engineering, School of Engineering and Technology, Sunway University, No.5, Jalan Universiti, Bandar Sunway, Petaling Jaya 47500, Selangor, Malaysia; khmo@sunway.edu.my; 3 Faculty of Engineering and Green Technology, Universiti Tunku Abdul Rahman, Jalan Universiti, Bandar Barat, Kampar 31900, Perak, Malaysia; yeapkh@utar.edu.my

**Keywords:** crumb rubber granules, polypropylene fibre, sustainable polymer composites, rubberised concrete, thermal insulation, mechanical properties

## Abstract

Disposing of waste tyres in landfills poses significant environmental hazards, making recycling a crucial alternative. Rubberised concrete has been found to exhibit lower density and better thermal insulation performance than conventional concrete. In order to maximise the potential of thermal insulation of rubberised concrete, this study investigates the mechanical and thermal properties of foamed rubberised polypropylene fibre concrete (FRPFC). FRPFC was produced using a mix of crumb rubber (CR) granules, polypropylene fibres, and foam, targeting a density of 800 kg/m^3^, with CR substituting sand at varying levels. Compressive strength, flexural strength, splitting tensile strength, and thermal conductivity of FRPFC were evaluated. The results demonstrate that increasing CR granule content enhances compressive strength due to reduced porosity from lower foam usage. For instance, compressive strength improved by 55% (2.64 to 4.10 MPa) as CR granule content increased from 0% to 80%. Similarly, flexural strength and splitting tensile strength increased by 55% (1.61 MPa to 2.49 MPa) and 39% (0.41 MPa to 0.57 MPa), respectively, when CR content rose from 0% to 100% at a water-to-cement ratio of 0.50. Furthermore, thermal conductivity decreased by 34% (0.3608 W/mK to 0.2376 W/mK) when sand was fully replaced with CR granules, showcasing improved thermal insulation. Statistical analysis using ANOVA confirmed that the crumb rubber content significantly influences the mechanical and thermal properties of FRPFC, with higher CR content (80% and 100%) leading to superior performance. These findings highlight FRPFC’s potential as an environmentally sustainable and thermally efficient construction material, contributing to enhanced mechanical properties compared to conventional foamed polypropylene fibre concrete.

## 1. Introduction

The development of vehicles has greatly improved convenience and quality of life. However, it has also posed significant environmental challenges. In addition to the emission of harmful gases that lead to air pollution and climate change, vehicles generate substantial waste when they reach the end of their service life. Among the most significant wastes are tyres, with approximately 1.5 billion being discarded annually [1]. Improper disposal of waste tyres in landfills or at illegal sites poses serious environmental risks, including rodent and insect infestations, increased fire hazards, as well as pollution due to leachate production and vector breeding [2]. As tyres are essential products, reducing their production is not a practical solution. Recycling waste tyres has emerged as the most viable approach to address this issue. For example, waste tyres can be converted into tyre-derived fuel, leveraging their high calorific value [3]. Additionally, incorporating waste tyres into concrete production provides another sustainable alternative. Depending on their processed form such as chips, granules, or powder, waste tyres can be used as aggregates in concrete [4,5].

In recent years, utilising scraps such as waste tyres, crushed glass, and crushed clay brick in concrete production has been an effective remedy to massively decrease the wastes, enhance the characteristics of concrete, cut down the consumption of natural aggregates, and minimise the emission of greenhouse gases arising from the production of concrete mixing materials [6]. The investigation has determined that the dry density of sustainable concrete decreases by 4%, 21.7%, and 31.7% upon the inclusion of crushed glass, clay brick, and waste tyres into the concrete in replacement of traditional aggregate, respectively. Waste rubber exhibits favourable outcomes in terms of sulphate, thermal, and impact resistance. Additionally, glass powder and finely crushed clay brick contributes to the enhancement of mechanical properties, and thermal resistance compared to the conventional aggregates in the concrete. Concrete that utilises waste tyres, often referred to as crumb rubber (CR), is termed rubberised concrete. Generally, the mechanical strength of rubberised concrete is substantially degraded. The deterioration can be mitigated by utilising rubber that has undergone heat pre-treatment at 200 °C for 2 h and subjected to 100% magnetised water treatment for 24 h. These treatments result in a 74% recovery in compressive strength and enhance impact resistance by 2.2 times at the onset of cracking [7]. In spite of the degradation effects in mechanical properties, the density of rubberised concrete is typically lower than that of conventional concrete, attributed to the low density of CR [8]. On top of that, rubberised concrete performs better than conventional concrete in several properties, including impact resistance, brittleness resistance, energy absorption, workability, permeability, thermal insulation and freeze–thaw resistance [9]. Moreover, a tipping fee will be provided to collect the waste tyres, which can help offset the concrete production cost. In order to maximise the potential of rubberised concrete, particularly in thermal insulation, investigation into porous or foamed rubberised polypropylene fibre concrete is drawing attention. This is because energy efficiency has always been an important sustainability criterion, and foamed concrete has been well recognised for its excellent thermal insulating properties, attributed to its cellular or porous microstructure [10]. However, the application of CR granules and foaming agent in concrete have been known to manifest a decline in the strength properties of concrete. Consequently, 0.6% of polypropylene fibre is incorporated into the concrete to improve its mechanical properties by alternating the fibre–matrix interface and force bridging mechanism across the crack of the concrete [11]. The polypropylene fibres that are drawn into the matrix delay the initiation of the degradation process, leading to reduced shrinkage and permeability and eventually minimising the expansion of the concrete [12]. It is reported that the inclusion of polypropylene fibre in foamed concrete with a density of 800 kg/m^3^ to 1500 kg/m^3^ increases the compressive strength by 5.6% to 33%, even though the workability of the concrete could be compromised [13]. Steel fibre was integrated into rubberised concrete to bolster its tensile and flexural strengths, toughness, and ductility, especially under elevated temperature conditions [14]. The research showcased a notable reduction in the risk of spalling and complexity in mechanical property evolution with temperature and heating duration.

Until today, research on lightweight foamed concrete incorporating both CR and polypropylene fibre has been fragmented and limited. The ductility and thermal properties of mortar, incorporating CR granules of different gradings and polypropylene fibre, were developed and studied [15]. The combination of rubber and fibre has greatly enhanced the failure mode, toughness, and flexural performance. Furthermore, analysis using the transient plane source method reveals notable improvements in thermal conductivity, thermal diffusivity, and heat capacity, indicative of enhanced heat insulation. Introducing 20% CR along with 1% fibre resulted in a 27.5% increase in flexural toughness compared to using 1% fibre alone. Moreover, incorporating 30% CR leads to a significant reduction in thermal conductivity by approximately 39.7%. Furthermore, the dynamic properties of conventional foamed concrete and foamed concrete amalgamated with recycled rubber granules and polypropylene fibre-modified rubber have recently been investigated [16]. The study demonstrated that the inclusion of polypropylene fibres significantly enhances the toughness of rubberised concrete by improving the fibre–matrix interface and crack-bridging mechanisms. Notably, the addition of rubber granules increased the impact resistance of the foamed concrete by 470.41% compared to conventional foamed concrete without rubber. This remarkable improvement can be attributed to the energy-absorbing properties of crumb rubber, which dissipates impact energy more effectively than traditional aggregates. Additionally, the combination of polypropylene fibres and rubber granules synergistically enhances the material’s ability to withstand dynamic loads, as the fibres delay crack propagation while the rubber granules absorb and redistribute stress. In comparison to foamed concrete with 1% polypropylene fibre alone, the inclusion of rubber granules further amplified the impact resistance by 27.5%, as reported in a related study [15]. This highlights the complementary role of rubber granules and polypropylene fibres in improving the mechanical performance of foamed concrete. While numerous studies have investigated the influence of rubber particles on the mechanical properties of foamed concrete, the synergistic effects of combining rubber granules with polypropylene fibres remain underexplored. This study aims to address this gap by comprehensively evaluating the mechanical, thermal, and durability properties of foamed rubberised polypropylene fibre concrete (FRPFC). For instance, one among these studies concludes that the integration of rubber particles in concrete demonstrates a downturn in compressive strength [4]. Concerning splitting tensile strength and flexural strength, the utilisation of rubber particles under a particular percentage contributes to a growth in both strengths. Despite the progress in research on individual components such as CR, polypropylene fibre, and foamed concrete, there is a lack of comprehensive studies that investigate the synergistic effects of incorporating both CR and polypropylene fibre into lightweight foamed concrete. While existing studies have demonstrated improvements in certain mechanical and thermal properties, there is a need to understand how these materials interact and influence each other when used together. Specifically, the combined effects of CR and polypropylene fibre on compressive strength, splitting tensile strength, flexural strength, and thermal insulation properties of lightweight foamed concrete remain largely unknown.

Utilising waste materials such as waste tyres, crushed glass, and the others in concrete production has been widely studied to address environmental challenges and enhance material properties. Therefore, this research builds on these efforts by introducing a novel approach that combines CR and polypropylene fibres in foamed concrete to explore their synergistic effects. Unlike previous studies that focus on these materials independently, this research investigates their combined impact on both mechanical strength and thermal insulation properties in foamed concrete. The aim of this study is to investigate the effect of incorporating high-volume CR granules on the mechanical properties and thermal insulation performance of FRPFC. The typical fine aggregate in foamed concrete, sand, is substituted with CR granules at varied percentages, with the targeted fresh density of the resulting FRPFC determined at 800 kg/m^3^. Compressive strength, flexural strength, splitting tensile strength, and thermal conductivity are examined for FRPFC specimens with varied CR replacement ratios, foam content, and water-to-cement ratios. This targeted density is optimised for non-structural applications, offering a sustainable and efficient alternative to conventional construction materials. Furthermore, the high-volume replacement of natural aggregates with CR granules not only enhances the performance of FRPFC but also aligns with environmental sustainability goals by addressing the critical issue of tyre waste management.

## 2. Materials and Methods

### 2.1. Materials

**Figure 1:**
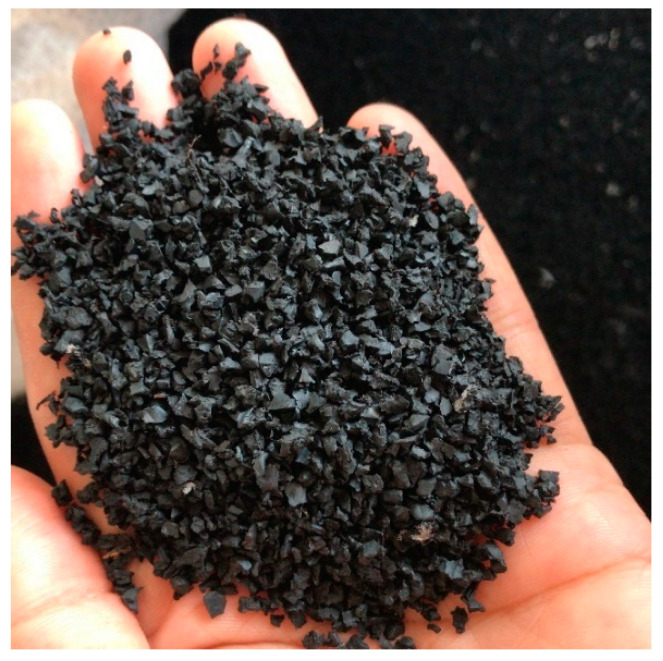
CR granules.

The binder used in this research was CEM I Ordinary Portland Cement (OPC) of strength class 42.5 N (Material, YTL Cement, Kuala Lumpur, Malaysia). Polypropylene fibre (Material, Mighty Shield Industries Sdn Bhd, Klang, Malaysia) was employed as an additive to the concrete to enhance certain properties. Its properties are shown in Table 1. Sand and CR granules (Material, Yong Fong Rubber Industries Sdn Bhd, Port Klang, Malaysia) were adopted as fine aggregate. The sand was sieved below 4.75 mm using a sieve (Instrument, Kensains Sdn Bhd, Petaling Jaya, Malaysia) and stored in an airtight container after 24 h oven drying at 100 °C. The CR granules were prepared using a smaller sieve size, between 1 mm and 4 mm, and they were used to partially or fully substitute the sand. The gradations of the sieved sand and CR granules are outlined in Table 2. Table 3 presents the properties, while Figure 1 displays the appearance of CR granules. To produce FRPFC, the pre-foaming method was used. SikaAER–50/50 (Reagent, Sika, Zurich, Switzerland), which is a high air entraining admixture for pumpable lightweight concrete, was employed as the foaming agent to generate the pre-form foam. The foaming process was conducted using a foam machine (Instrument, AC Mall, Pahang, Malaysia) with a density of 45 kg/m^3^. Tap water was used throughout the experimental work.

### 2.2. Mix Proportion

In this research, low-density FRPFC specimens were produced to ensure their lightweight characteristics, so that those specimens exhibiting desired mechanical and thermal insulating properties could be employed in subsequent studies, including the use of FRPFC material as the core of a sandwich wall panel system in non-structural applications. Irrespective of this, a high replacement ratio of CR granules was implemented because this research aimed to formulate an FRPFC mix that maximises the utilisation of waste materials while simultaneously meeting the criteria for specific building applications. Table 4 shows the mixed proportion of the FRPFC specimens investigated in this work. Each mix was designed to produce FRPFC with an 800 kg/m^3^ target density. The proportion of cement and fine aggregate was fixed at 1:1. In total, 0.6% of polypropylene fibre was added to each concrete mix. Moreover, the fine aggregate sand was partially or fully substituted by the CR granules, by volume, at various percentages, i.e., 0%, 20%, 40%, 60%, 80%, and 100%. The designation of the specimen is (Foamed Rubberised Polypropylene Fibre Concrete) FRPFCX:Y; X represents the w/c ratio, while Y indicates the sand replacement percentage with the CR granules.

### 2.3. Sample Preparation

To prepare the FRPFC, firstly, cement, sand, polypropylene fibre and/or CR granules were weighed accordingly and mixed in a planetary mixer to obtain a homogenous dry mixture. Then, water was added, followed by the addition of the pre-formed foam. The pre-formed foam was prepared using a foam generator by diluting 1 part foaming agent with 20 parts of water. The produced foam was introduced into the slurry until the fresh density achieved 800 ± 20 kg/m^3^; the fresh density of FRPFC was measured with the aid of a 1 L container complying to the standard ASTM C138 [17]. Upon mixing, the fresh FRPFC was immediately poured into respective moulds and subject to air curing for 24 h. Once the concrete specimens had hardened and were demoulded, they were transferred to a water tank for curing until the 28th day.

### 2.4. Testing Methods

**Figure 2:**
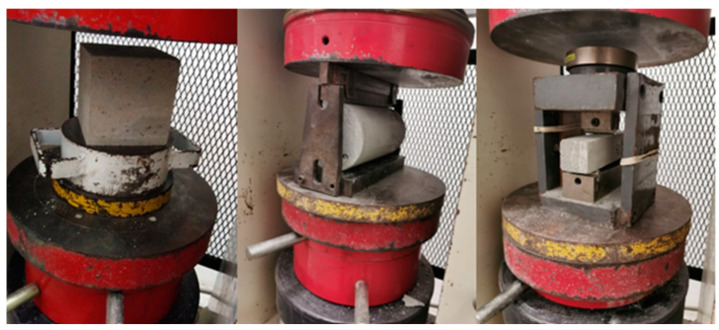
Setups of the mechanical properties tests.

The mechanical properties of FRPFC, namely, compressive strength, splitting tensile strength, and flexural strength, were measured in accordance with BS EN1230-3 [18], ASTM C496 [19], and ASTM C293 [20], respectively. FRPFC cube of 100 mm × 100 mm × 100 mm was employed for the compressive strength test, while the splitting tensile and flexural strengths tests were conducted using cylindrical FRPFC and FRPFC prism of 100 mm dia. × 200 mm and 40 mm × 40 mm × 160 mm, respectively. The respective setups of the mechanical properties tests are illustrated in Figure 2.

**Figure 3:**
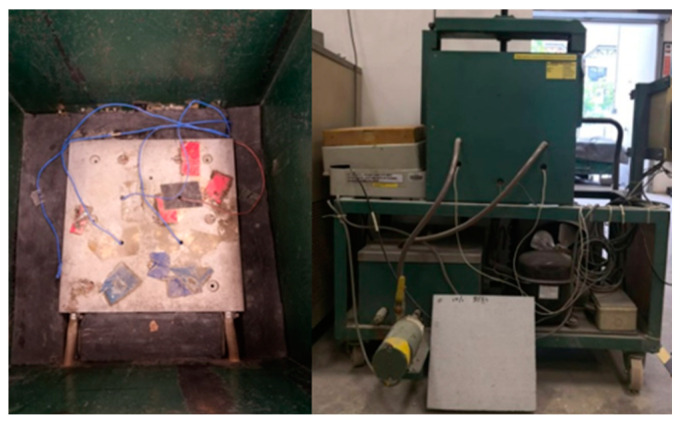
Thermal transmission properties testing machine.

For the thermal conductivity test, the FRPFC of 300 mm × 300 mm × 50 mm was set up between 2 plates—hot and cold plates. The experiment setup is shown in Figure 3 and was conducted in accordance with the ASTM C 177 [21]. The temperature changes at the hot and cold plates were measured and recorded every hour using sensors until the steady-state heat flux was attained.

All the tests were conducted using the FRPFC specimens that had been water-cured for 28 days. Three FRPFC specimens were employed for each test, and the respective average results were reported.

## 3. Results and Discussions

### 3.1. Fresh and Hardened Densities

**Figure 4:**
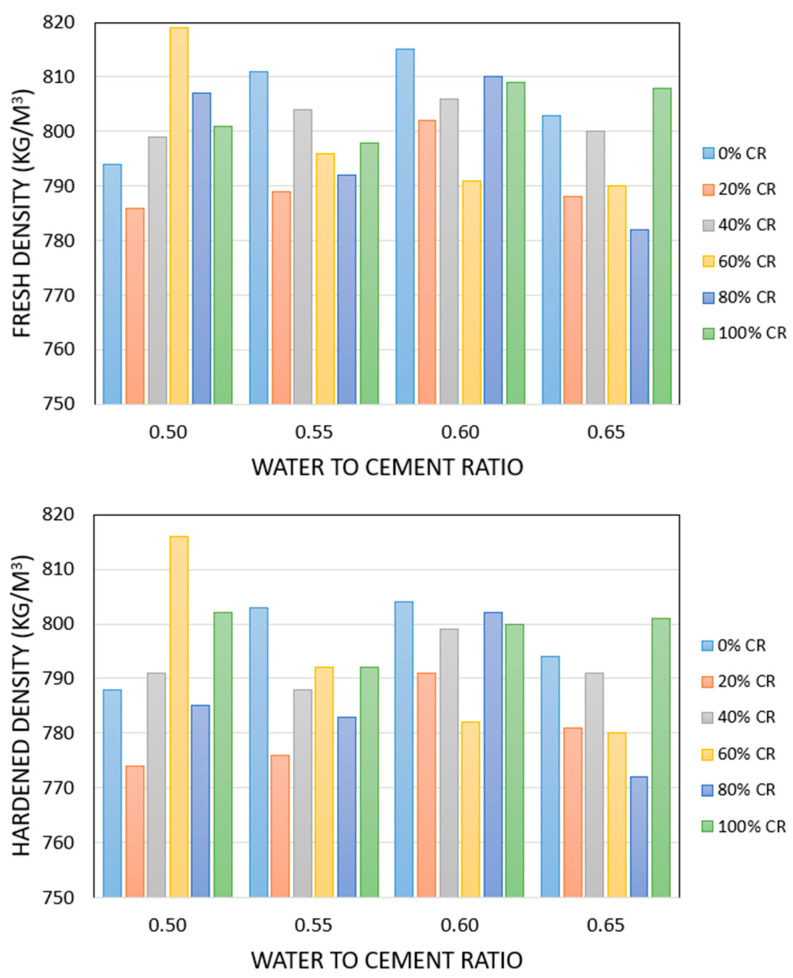
Fresh and hardened densities of FRPFC specimens.

The fresh and hardened densities of FRPFC specimens are outlined in Table 5 and graphically presented in Figure 4. The fresh density was controlled within the range of 800 ± 20 kg/m^3^.

### 3.2. Compressive Strength

**Figure 5:**
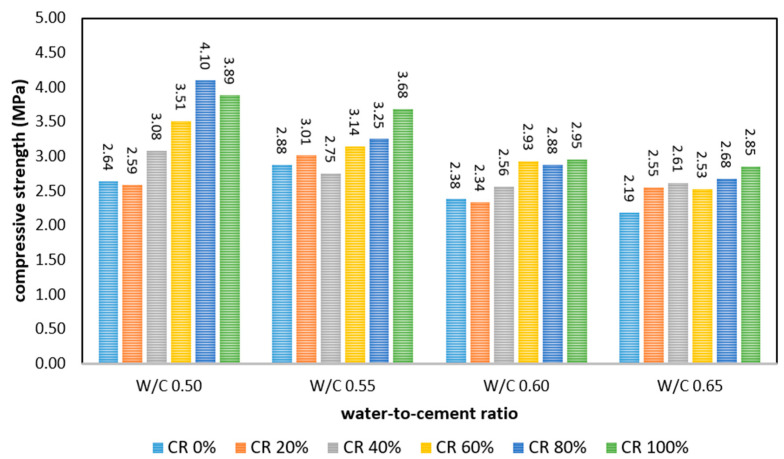
Compressive strength of FRPFC.

Figure 5 shows the compressive strength of FRPFC with different CR granule substitution percentages as well as varied water-to-cement (w/c) ratios. In general, compressive strength was inversely proportional to the percentage of the w/c ratio [22]. The reduction in strength was likely attributed to the dilution effect associated with the elevated water content in the concrete mixture. A high w/c ratio decreases the proportion of cementitious material, which plays a vital role in developing concrete strength during the hardening stage, in addition to reducing the strength of individual aggregates. Specifically, when the w/c ratio increased from 0.50 to 0.65, the strengths of FRPFC0.65:0, FRPFC0.65:20, FRPFC0.65:40, FRPFC0.65:60, FRPFC0.65:80, and FRPFC0.65:100 were reduced by 0.45 MPa (17%), 0.04 MPa (2%), 0.47 MPa (15%), 0.98 MPa (28%), 1.42 MPa (35%), and 1.04 MPa (27%), respectively.

Conversely, at a constant w/c ratio, the increase in CR granule content in the FRPFC specimens did not degrade compressive strength. Traditionally, the substitution of sand with CR is expected to reduce strength [23]. However, this study investigated FRPFC specimens with similar fresh densities. Since CR granules are significantly lighter than sand, the initial fresh density of FRPFC, without foaming, was generally lower, necessitating less pre-formed foam to achieve a target density of 800 kg/m^3^. As a result, FRPFC achieved a relatively compact microstructure, contributing to higher compressive strength, which aligns with previous reports [4,24].

**Figure 6:**
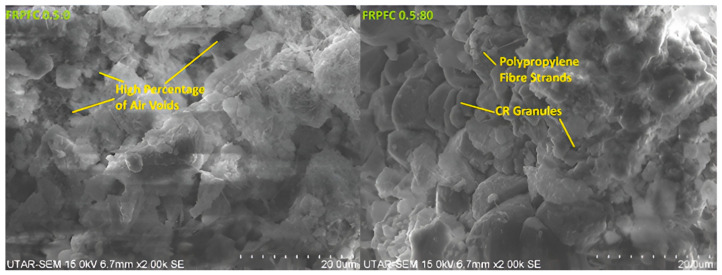
Microstructural illustration of specimens FRPFC 0.5:0 and FRPFC 0.5:80.

Among the investigated specimens, FRPFC0.50:80 (with 80% CR granules as fine aggregate) exhibited the highest compressive strength of 4.10 MPa. This can be attributed to a portion of air voids being filled by the CR granules as the replacement percentage increased to 80%, which is graphically represented in Figure 6. This filling effect reduced the number of air bubbles per unit cross-sectional area, thereby enhancing compressive strength [16]. Additionally, the dispersion of polypropylene fibre strands within the FRPFC matrix was assessed using optical microscopy and scanning electron microscopy (SEM), revealing that the polypropylene fibres were uniformly distributed throughout the matrix with minimal clustering, as shown in Figure 6. This uniform dispersion is critical, as it ensures the fibres can effectively bridge microcracks, thereby contributing to the enhanced tensile strength and ductility of FRPFC.

The interfacial bond strength between the polypropylene fibres and the cement paste was also a key factor in the material’s performance. Pull-out tests showed an average bond strength of 1.2 MPa, which is attributed to the rough surface texture of the polypropylene fibres that enhances mechanical interlocking with the cement matrix. SEM analysis confirmed that the polypropylene fibres are well bonded to the cement paste, with no visible gaps or debonding. This strong interfacial bond is essential for effective stress transfer across cracks and significantly enhances both the tensile strength and ductility of FRPFC.

Furthermore, SEM analysis in related studies has shown that CR particles create a rougher interfacial transition zone (ITZ) with the cement paste, improving mechanical interlocking and supporting enhanced flexural performance [7]. However, compressive strength decreased beyond the 80% substitution level, dropping from 4.10 MPa to 3.89 MPa. Although this reduction was minimal, it was primarily attributed to insufficient water content caused by the high volume of CR granules at 100%.

Increasing the water content to compensate for reduced workability at 100% CR granules can have significant drawbacks. Higher w/c ratios reduce the cementitious material available for bonding, weakening the matrix and increasing porosity. This dilution effect outweighs the potential strength increase from CR granules filling air voids, as evidenced by the lower compressive strength of FRPFC with higher w/c ratios. For instance, the compressive strength of FRPFC at a w/c ratio of 0.65 was consistently lower compared to 0.50 at similar CR granule levels, emphasising that the disadvantages of adding water surpass the benefits of increasing CR content beyond 80%.

### 3.3. Flexural Strength

**Figure 7:**
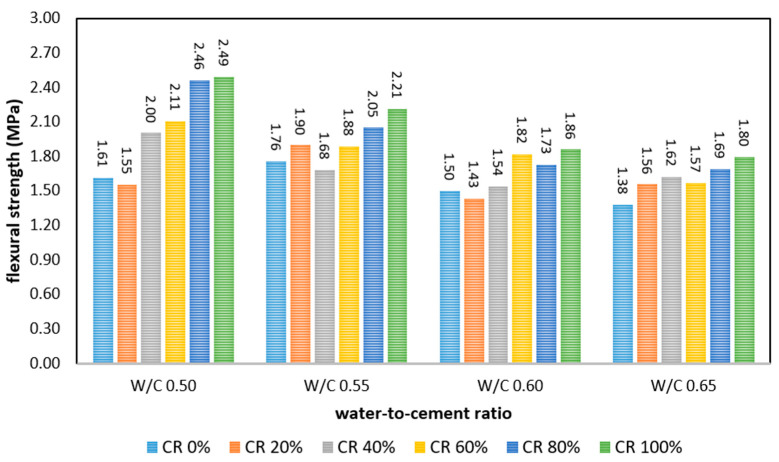
Flexural strength of FRPFC.

The flexural strength of FRPFC with different CR proportions and moisture contents is illustrated in Figure 7. Basically, the flexural strength of FRPFC dropped with the increased w/c ratio, from 0.50 to 0.65. Nevertheless, such a trend was not observed on the FRPFC with a CR content of 20%. The change in the flexural strength as the w/c ratio increased was rather irregular. Among them, FRPFC0.55:20 was reported to possess the greatest flexural strength, 1.90 MPa. When the w/c ratio was varied from 0.50 to 0.65, the strength of FRPFC0.65:0, FRPFC0.65:20, FRPFC0.65:40, FRPFC0.65:60, FRPFC0.65:80, and FRPFC0.65:100 approximately declined by 0.23 MPa (14%), −0.01 MPa (0%), 0.38 MPa (19%), 0.54 MPa (26%), 0.77 MPa (31%), and 0.69 MPa (28%), respectively.

Subsequently, at a constant w/c ratio, the relationship between the flexural strength and the CR replacement ratio is ascertained. It was observed that the flexural strength of FRPFC0.5 was increased with the addition of CR granule content, except for a slight reduction observed from FRPFC0.5:20. The reduction might be attributed to the synergistic effect of the high foam volume and usage of CR granules.

At a constant w/c ratio, the relationship between flexural strength and CR replacement ratio indicated that the flexural strength of FRPFC0.5 increased with the addition of CR granules, except for a slight reduction observed with FRPFC0.5:20, potentially attributed to the synergistic effect of high foam volume and CR granules. Among the specimens, FRPFC with full sand substitution with CR granules exhibited the highest flexural strength of 2.49 MPa. This significant increase can be attributed to the elastic nature of CR granules, which absorb energy under compressive or flexural loads. Their incorporation leads to a denser microstructure with reduced porosity and improved pore structure, providing a more continuous load-bearing path and stress concentrations minimisation that enhances the flexural strength.

**Figure 8:**
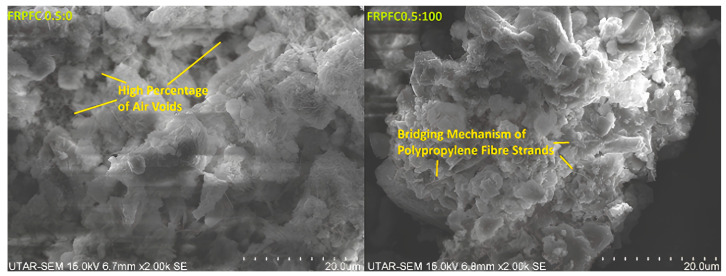
Microstructural illustration of specimens FRPFC 0.5:0 and FRPFC 0.5:100.

When combined with polypropylene fibre, the rubber particles effectively reduce stress concentrations at crack tips, while the fibre prevents catastrophic failure. This combined mechanism explains the observed 55% increase in flexural strength at 100% CR substitution. In contrast, FRPFC with a lower CR granule content, including the control with zero CR inclusion, tended to have a more porous microstructure, as depicted in Figure 8. Furthermore, the infusion of a substantial volume of air bubbles into the concrete undermined the bridging mechanism imposed by the polypropylene fibre strands [25]. Throughout the concrete mixing process, a significant quantity of small air bubbles congregated around the polypropylene fibre strands, amalgamating into larger air bubbles. Consequently, this diminished the flexural load capacity within the plane containing a high percentage of air voids. A similar trend was reported, where the flexural strength increased when the foam volume was decreased [4].

### 3.4. Splitting Tensile Strength

**Figure 9:**
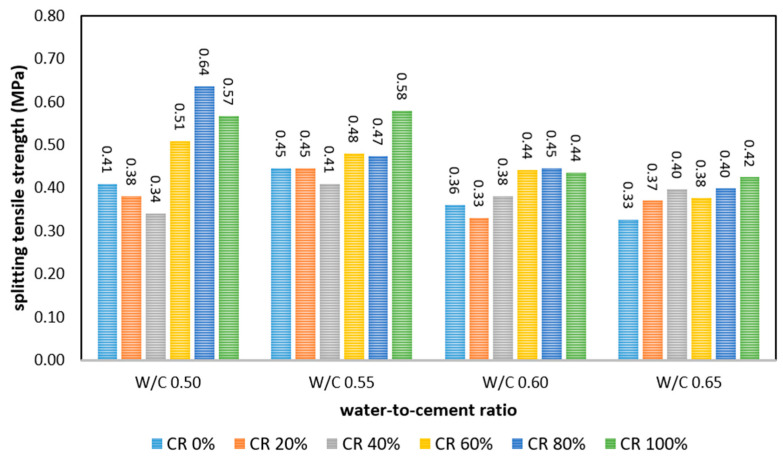
Splitting tensile strength of FRPFC.

The relationship between the splitting tensile strength of FRPFC with multiple CR granule replacement ratio and the w/c ratio is depicted in Figure 9. Generally, the trend of strength–w/c ratio relationship was the same as those of compressive strength and flexural strength. As the water content of FRPFC rose from 0.50 to 0.65, the splitting tensile strength decayed. This was because of the compositions of binder, aggregates, as well as polypropylene fibre, as the concrete additives were much lower in FRPFC with high moisture content. Besides the dilution effect, workability was also one of the key factors influencing the strength properties of FRPFC. At a low w/c ratio of 0.50, the workability of FRPFC was still not compromised; thus, strength decrease associated with the workability was not spotted. When the w/c ratio was increased from 0.50 to 0.65, the strengths of FRPFC0.65:0, FRPFC0.65:20, FRPFC0.65:40, FRPFC0.65:60, FRPFC0.65:80, and FRPFC0.65:100 were weakened by 0.08 MPa (20%), 0.01 MPa (3%), −0.06 MPa (−17%), 0.13 MPa (26%), 0.24 MPa (37%), and 0.15 MPa (25%), respectively.

Next, the behavior of splitting tensile strength-CR granule content was investigated. The splitting tensile strength of FRPFC 0.50 decreased at low CR granule content levels of 20% and 40%. Although the foam dosage decreased with an increase in the crumb rubber replacement ratio from 0% to 40%, the combined effects of weak interfacial bonding and reduced matrix stiffness caused by the interplay between foam and crumb rubber resulted in an initial deterioration in splitting tensile strength.

Nonetheless, the splitting tensile strength of FRPFC increased when the CR granules were beyond 40% and reached the maximum when the CR granule content was 80%. The increment was because a higher volume of CR granules was used to substitute the sand. As the use of foam decreased, its weakening effect the FRPFC also decreased. CR granules ranging from 1 to 4 mm in particle size aided in bridging cracks as the applied load progressed and ultimately delayed the failure. The nature of failure was altered from brittle to flexural [4]. Additionally, it was also reported that the splitting tensile of CR concrete without foaming decreased with the 20% CR granules substitution level [26]. Porosimetry analysis of FRPFC 0.50:0 and FRPFC 0.50:80 further supports this trend. As shown in the SEM images in Figure 6, FRPFC 0.50:0 exhibited a high percentage of air voids, which contributed to reduced matrix density and lower strength. In contrast, the microstructure of FRPFC 0.50:80 displayed well-distributed CR granules and polypropylene fibre strands, leading to a more interconnected and compact matrix. The presence of CR granules acted as a crack-bridging mechanism, improving stress distribution and delaying fracture propagation. Hence. When CR granule replacement level was beyond 80%, generally, the splitting tensile strength of FRPFC with various w/c ratios decreased.

### 3.5. Thermal Insulation Properties

**Figure 10:**
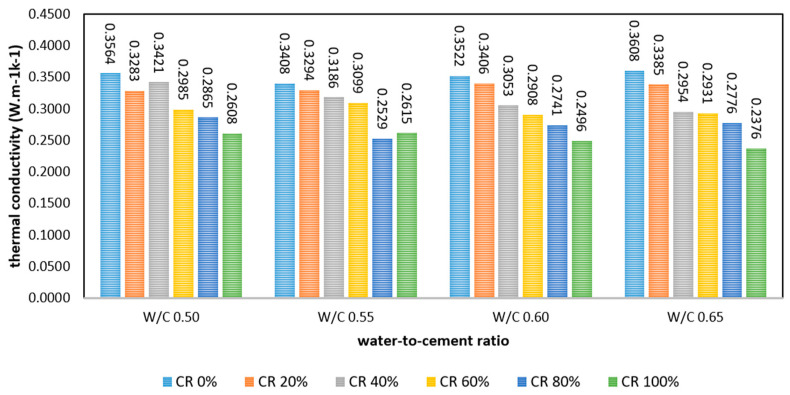
Thermal conductivity of FRPFC.

The thermal conductivity of FRPFC produced with various w/c ratios and substitution levels of CR granules is shown in Figure 10. The thermal conductivity of FRPFC was dependent on the individual thermal insulating properties of each concrete mixing material and the microstructure of the FRPFC. Based on the data and results, the effect of the w/c ratio on the thermal conductivity of FRPFC was rather ambiguous and anomalous. For all CR proportions, the change in thermal conductivity with the increased water content from 0.50 to 0.65 was minimal.

**Figure 11:**
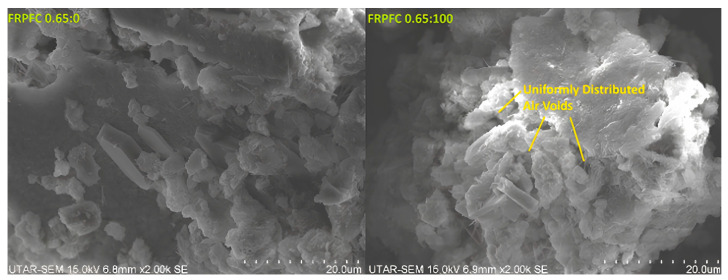
Microstructural illustration of specimens FRPFC 0.65:0 and FRPFC 0.65:100.

However, for a given water-to-cement (w/c) ratio, such as 0.65, the thermal conductivity of FRPFC decreased remarkably as the level of CR granule substitution increased. Specifically, as the CR granule substitution level rose from 0% to 100%, the thermal conductivity of FRPFC specimens at various replacement levels (FRPFC0.65:20, FRPFC0.65:40, FRPFC0.65:60, FRPFC0.65:80, and FRPFC0.65:100) was reduced by 0.0223 Wm^−1^K^−1^ (6%), 0.0654 Wm^−1^K^−1^ (18%), 0.0677 Wm^−1^K^−1^ (19%), 0.0832 Wm^−1^K^−1^ (23%), and 0.1232 Wm^−1^K^−1^ (34%), respectively. This decline in thermal conductivity can primarily be attributed to the insulating properties, non-wetting characteristics, and elevated porosity of the CR particles within the concrete matrix [27]. Just as observed in PP/MMT composite foams, where smaller, closed-cell pores (2–4 μm) create a similar hierarchical pore structure, CR granules effectively fill larger voids while foam introduces porosity, optimising insulation without compromising strength [28]. The closed-cell pore structure in FRPFC with high CR content contributes to its lower thermal conductivity because the spherical pores act as insulating air pockets, reducing heat transfer through the material. As CR demonstrated superior insulating and porosity characteristics compared to sand, substituting CR for sand enhanced the overall insulating properties of the final concrete product. Furthermore, the non-polar nature of the rubber particles tends to entrap air on their surfaces, leading to a more uniform distribution of induced air voids throughout the concrete matrix, as illustrated in Figure 11. This uniform distribution improves thermal insulation by creating a network of air pockets that inhibits heat transfer [29]. Thus, partially or fully replacing sand with CR granules effectively reduced thermal conductivity, even though the pre-formed foam’s pores were somewhat minimised.

### 3.6. Statistical Interpretation of the Data

To ensure the robustness and reliability of the experimental results, a comprehensive statistical analysis was conducted on the mechanical and thermal properties of FRPFC. The analysis aimed to determine whether the observed differences in compressive strength, splitting tensile strength, flexural strength, and thermal conductivity across different CR content groups were statistically significant. Statistical methods, including analysis of variance (ANOVA) and Tukey’s honestly significant difference (HSD) post-hoc tests, were employed to evaluate the impact of CR content on the properties of FRPFC. The results of the statistical analysis are presented in this section, providing insights into the significance of CR content in optimising the performance of FRPFC for sustainable construction applications. The data of the six CR groups of FRPFC are presented in Table 6.

The ANOVA test partitions the total variability in the data into between-group variability (due to differences in CR content) and within-group variability (due to random variation). The F-value and *p*-value were calculated to assess the significance of the between-group differences. A *p*-value < 0.05 was considered statistically significant, indicating that the CR content had a significant impact on the property being analyzed. The results of the ANOVA tests are presented in Table 7, along with the degrees of freedom (df), sum of squares (SS), mean square (MS), F-value, and *p*-value for each property.

It is shown that the CR content has a statistically significant impact on the compressive strength, splitting tensile strength, flexural strength, and thermal conductivity of FRPFC. For all properties, the *p*-values are <0.05, indicating that the differences between the six CR content groups (0%, 20%, 40%, 60%, 80%, and 100%) are statistically significant. Specifically, the F-values for compressive strength (8.45, *p* = 0.0002), splitting tensile strength (4.56, *p* = 0.0061), flexural strength (6.78, *p* = 0.0011), and thermal conductivity (12.34, *p* = 0.0001) confirm that the CR content significantly influences these properties. Post-hoc analysis using HSD testing was then performed to identify which specific CR content groups differed significantly from each other, and the results are tabulated in Table 8.

The ANOVA results reveal that the CR content has a statistically significant impact on the compressive strength, splitting tensile strength, flexural strength, and thermal conductivity of FRPFC. For compressive strength, the 80% and 100% CR content groups exhibit significantly higher values compared to the 0%, 20%, and 40% groups, likely due to the reduced porosity and improved microstructure achieved by incorporating higher volumes of CR granules. Similarly, for splitting tensile strength, the 80% and 100% CR content groups show significantly higher values compared to the 0% and 20% groups, attributed to the crack-bridging effect of CR granules. For flexural strength, the 80% and 100% CR content groups also demonstrate significantly higher values compared to the 0% and 20% groups, owing to the improved ductility and energy absorption provided by the CR granules. For thermal conductivity, the 80% and 100% CR content groups exhibit significantly lower values compared to the 0%, 20%, 40%, and 60% groups, due to the air-entrapping nature of CR granules, which creates a network of insulating air pockets that hinder heat transfer. These findings highlight the importance of CR content in optimising the mechanical and thermal properties of FRPFC, with higher CR content (80% and 100%) generally leading to superior performance. This provides a statistically robust basis for selecting the optimal CR content for specific applications, balancing mechanical properties, thermal insulation, and sustainability goals.

## 4. Conclusions

In conclusion, FRPFC, particularly when enriched with a higher density of CR granules, outperforms conventional foamed polypropylene fibre concrete in both mechanical strength and thermal conductivity. This is because a high volume of foam is needed to achieve a satisfactory lightweight nature. Thus, foamed polypropylene fibre concrete is highly porous. On the other hand, FRPFC can achieve similar lightweight characteristics as foamed concrete but with lesser porosity. This is because the combination effects of foam and CR granules reduce the density of FRPFC. This study used CR granules to replace the natural fine aggregate, sand. Since the density of CR granules is much less than the sand, less foam is needed to reduce the density of FRPFC to 800 kg/m^3^. As a result of this investigation, the following conclusions can be drawn:

1. FRPFC with a high volume of pre-formed foam (lesser CR granule content, i.e., <20%) exhibits a weaker mechanical strength.
2. FRPFC with a 0.50 w/c ratio and 80% CR granule substitution level exhibited the highest compressive strength, 4.10 MPa.
3. Increasing the CR granule content in FRPFC with the moisture content between 0.50 and 0.65 improved compressive strength, splitting tensile strength and flexural strength of FRPFC. This improvement is primarily attributed to the reduced foam dosage as CR replaces sand, leading to a less porous and denser microstructure. Additionally, the crack-bridging ability of CR granules further enhanced the mechanical performance of the FRPFC.
4. The thermal conductivity of FRPFC remained relatively constant when the water-to-cement ratio changed within the range of 0.5 to 0.65. Meanwhile, the thermal conductivity of FRPFC was decreased with increasing CR granule portion. FRPFC0.80:100 possessed the lowest thermal conductivity, 0.2376 Wm^−1^K^−1^.

The statistical analysis further supports these findings, demonstrating that the crumb rubber content has a statistically significant impact on the mechanical and thermal properties of FRPFC. The ANOVA results reveal that higher CR content (80% and 100%) significantly enhances compressive strength, splitting tensile strength, and flexural strength, while also reducing thermal conductivity. These results confirm that the optimisation of CR content is critical for achieving the desired balance of mechanical performance and thermal insulation in FRPFC.

The main application of the FRPFC is to be adopted as the core material in a sandwich wall panel system for building construction. The core material must possess adequate mechanical strength to meet building safety and structural criteria while offering superior thermal insulation to ensure energy efficiency and indoor comfort for inhabitants. This research confirms that FRPFC fulfills these requirements, making it a viable, sustainable, and efficient material for modern construction applications.

## 5. Recommendations

This study is centered on the concrete mix design, incorporating varying foam and CR granules to achieve FRPFC with target densities of approximately 800 kg/m^3^. However, future studies should encompass a wider range of concrete target densities and the mixing compositions of CR granules, foam, sand and cement, in search of an FRPFC material that suits being adopted as the core of the sandwich wall panel system. Moreover, exploring rubber particle pre-treatments and the incorporation of other fibre additives presents a potential approach for enhancing FRPFC properties. Lastly, conducting energy-dispersive X-ray analysis (EDX) for FRPFC with distinct mixing parameters could provide comprehensive insights into the morphology, chemical composition and crystallographic information, all of which affect the mechanical and thermal insulating properties of FRPFC.

## Figures and Tables

**Table 1:** Properties of polypropylene fibre.

Properties	Result
Fibre type	Single fibre
Length	12 mm
Diameter	0.034 mm
Density	0.91 g/mm^3^
Ultimate tensile strength	700 MPa
Modulus of elasticity	2.8 GPa

**Table 2:** Gradations of sand and CR granules as the fine aggregate.

Sieve Size	Cumulative Percentage Passing (%)	
	Sand	CR Granules
10 mm	100	100
4.75 mm	100	100
2.36 mm	86	48
1.18 mm	71	8
600 µm	54	0
300 µm	32	0
150 µm	10	0

**Table 3:** Properties of CR granules.

Physical Properties	Result	Chemical Composition	Content
Density	0.95 g/mm^3^	Styrene butadiene rubber (SBR)	48%
Ultimate tensile strength	9 MPa	Carbon black	31%
Elongation at break	150%	Acetone extract	15%
Hardness shore	64	Ash	2%
Particle size range	1 mm to 4 mm	Residue chemical balance	4%

**Table 4:** Mix proportion of concrete specimen sets.

Designation	Material (kg/m^3^)						W/C	Target Density (kg/m^3^)
	Cement	Sand	Water	Polypropylene Fibre	Crumb Rubber	Foam		
FRPFC0.5:0	306.85	306.85	153.42	4.60	0.00	28.27	0.50	800
FRPFC0.5:20	322.26	257.81	161.13	4.61	26.75	27.45		
FRPFC0.5:40	339.30	203.58	169.65	4.61	56.34	26.53		
FRPFC0.5:60	358.24	143.29	179.12	4.62	89.22	25.51		
FRPFC0.5:80	379.42	75.88	189.71	4.63	125.99	24.37		
FRPFC0.5:100	403.26	0.00	201.63	4.63	167.39	23.09		
FRPFC0.55:0	300.97	300.97	165.54	4.60	0.00	27.91	0.55	
FRPFC0.55:20	315.78	252.63	173.68	4.61	26.22	27.08		
FRPFC0.55:40	332.13	199.28	182.67	4.62	55.15	26.17		
FRPFC0.55:60	350.25	140.10	192.64	4.62	87.23	25.15		
FRPFC0.55:80	370.47	74.09	203.76	4.63	123.03	24.02		
FRPFC0.55:100	393.17	0.00	216.24	4.64	163.20	22.75		
FRPFC0.60:0	295.32	295.32	177.19	4.61	0.00	27.56	0.60	
FRPFC0.60:20	309.56	247.65	185.74	4.61	25.70	26.73		
FRPFC0.60:40	325.25	195.15	195.15	4.62	54.00	25.82		
FRPFC0.60:60	342.62	137.05	205.57	4.62	85.33	24.81		
FRPFC0.60:80	361.94	72.39	217.17	4.63	120.19	23.68		
FRPFC0.60:100	383.58	0.00	230.15	4.64	159.22	22.42		
FRPFC0.65:0	289.87	289.87	188.42	4.61	0.00	27.23	0.65	
FRPFC0.65:20	303.58	242.87	197.33	4.61	25.20	26.40		
FRPFC0.65:40	318.66	191.20	207.13	4.62	52.91	25.49		
FRPFC0.65:60	335.31	134.12	217.95	4.63	83.51	24.48		
FRPFC0.65:80	353.80	70.76	229.97	4.63	117.49	23.36		
FRPFC0.65:100	374.44	0.00	243.38	4.64	155.43	22.11		

**Table 5:** Fresh and hardened densities of FRPFC specimens.

Designation	Fresh Density (kg/m^3^)	Hardened Density (kg/m^3^)
FRPFC0.5:0	794	788
FRPFC0.5:20	786	774
FRPFC0.5:40	799	791
FRPFC0.5:60	819	816
FRPFC0.5:80	807	785
FRPFC0.5:100	801	802
FRPFC0.55:0	811	803
FRPFC0.55:20	789	776
FRPFC0.55:40	804	788
FRPFC0.55:60	796	792
FRPFC0.55:80	792	783
FRPFC0.55:100	798	792
FRPFC0.60:0	815	804
FRPFC0.60:20	802	791
FRPFC0.60:40	806	799
FRPFC0.60:60	791	782
FRPFC0.60:80	810	802
FRPFC0.60:100	809	800
FRPFC0.65:0	803	794
FRPFC0.65:20	788	781
FRPFC0.65:40	800	791
FRPFC0.65:60	790	780
FRPFC0.65:80	782	772
FRPFC0.65:100	808	801

**Table 6:** Statistical data of the six CR groups of FRPFC.

Crumb Rubber Proportion (%)	Designation	Compressive Strength (MPa)	Splitting Tensile Strength (MPa)	Flexural Strength (MPa)	Thermal Conductivity (Wm^−1^K^−1^)
0	FRPFC0.5:0	2.64	0.41	1.61	0.3564
	FRPFC0.55:0	2.88	0.45	1.76	0.3408
	FRPFC0.60:0	2.38	0.36	1.50	0.3522
	FRPFC0.65:0	2.19	0.33	1.38	0.3608
20	FRPFC0.5:20	2.59	0.38	1.55	0.3283
	FRPFC0.55:20	3.01	0.45	1.90	0.3294
	FRPFC0.60:20	2.34	0.33	1.43	0.3406
	FRPFC0.65:20	2.55	0.37	1.56	0.3385
40	FRPFC0.5:40	3.08	0.34	2.00	0.3421
	FRPFC0.55:40	2.75	0.41	1.68	0.3186
	FRPFC0.60:40	2.56	0.38	1.54	0.3053
	FRPFC0.65:40	2.61	0.40	1.62	0.2954
60	FRPFC0.5:60	3.51	0.51	2.11	0.2985
	FRPFC0.55:60	3.14	0.48	1.88	0.3099
	FRPFC0.60:60	2.93	0.44	1.82	0.2908
	FRPFC0.65:60	2.53	0.38	1.57	0.2931
80	FRPFC0.5:80	4.10	0.64	2.46	0.2865
	FRPFC0.55:80	3.25	0.47	2.05	0.2529
	FRPFC0.60:80	2.88	0.45	1.73	0.2741
	FRPFC0.65:80	2.68	0.40	1.69	0.2776
100	FRPFC0.5:100	3.89	0.57	2.49	0.2608
	FRPFC0.55:100	3.68	0.58	2.21	0.2615
	FRPFC0.60:100	2.95	0.44	1.86	0.2496
	FRPFC0.65:100	2.85	0.42	1.80	0.2376

**Table 7:** ANOVA results.

Property	Source of Variation	Degrees of Freedom (df)	Sum of Squares (SS)	Mean Square (MS)	F-Value	*p*-Value
Compressive Strength	Between Groups	5	5.23	1.05	8.45	0.0002
	Within Groups	18	2.24	0.12		
	Total	23	7.47			
Splitting Tensile Strength	Between Groups	5	0.12	0.024	4.56	0.0061
	Within Groups	18	0.095	0.0053		
	Total	23	0.215			
Flexural Strength	Between Groups	5	1.45	0.29	6.78	0.0011
	Within Groups	18	0.77	0.043		
	Total	23	2.22			
Thermal Conductivity	Between Groups	5	0.045	0.009	12.34	0.0001
	Within Groups	18	0.013	0.0007		
	Total	23	0.058			

**Table 8:** ANOVA results with significant comparisons.

Property	F-Value	*p*-Value	Significance	Significant Comparisons (Tukey’s HSD)
Compressive Strength	8.45	0.0002	Significant	0% vs. 80%: Mean Difference = 1.46 MPa, *p* = 0.0001 0% vs. 100%: Mean Difference = 1.25 MPa, *p* = 0.0018 20% vs. 80%: Mean Difference = 1.51 MPa, *p* = 0.0001 20% vs. 100%: Mean Difference = 1.30 MPa, *p* = 0.0009 40% vs. 80%: Mean Difference = 1.02 MPa, *p* = 0.0102 40% vs. 100%: Mean Difference = 0.81 MPa, *p* = 0.0398
Splitting Tensile Strength	4.56	0.0061	Significant	0% vs. 80%: Mean Difference = 0.23 MPa, *p* = 0.0198 0% vs. 100%: Mean Difference = 0.20 MPa, *p* = 0.0395 20% vs. 80%: Mean Difference = 0.21 MPa, *p* = 0.0297 20% vs. 100%: Mean Difference = 0.18 MPa, *p* = 0.0492
Flexural Strength	6.78	0.0011	Significant	0% vs. 80%: Mean Difference = 0.88 MPa, *p* = 0.0009 0% vs. 100%: Mean Difference = 0.81 MPa, *p* = 0.0018 20% vs. 80%: Mean Difference = 0.91 MPa, *p* = 0.0008 20% vs. 100%: Mean Difference = 0.84 MPa, *p* = 0.0015
Thermal Conductivity	12.34	0.0001	Significant	0% vs. 80%: Mean Difference = 0.0699 W/mK, *p* = 0.0001 0% vs. 100%: Mean Difference = 0.1188 W/mK, *p* = 0.0001 20% vs. 80%: Mean Difference = 0.0542 W/mK, *p* = 0.0018 20% vs. 100%: Mean Difference = 0.1031 W/mK, *p* = 0.0001 40% vs. 80%: Mean Difference = 0.0556 W/mK, *p* = 0.0012 40% vs. 100%: Mean Difference = 0.1045 W/mK, *p* = 0.0001 60% vs. 80%: Mean Difference = 0.0124 W/mK, *p* = 0.0398 60% vs. 100%: Mean Difference = 0.0613 W/mK, *p* = 0.0018

## Data Availability

The datasets presented in this article are not readily available because the data are part of an ongoing study.
